# Regulation of Energy Metabolism by the Extracytoplasmic Function (ECF) σ Factors of *Arcobacter butzleri*

**DOI:** 10.1371/journal.pone.0044796

**Published:** 2012-09-18

**Authors:** Irati Martinez-Malaxetxebarria, Rudy Muts, Linda van Dijk, Craig T. Parker, William G. Miller, Steven Huynh, Wim Gaastra, Jos P. M. van Putten, Aurora Fernandez-Astorga, Marc M. S. M. Wösten

**Affiliations:** 1 Department of Infectious Diseases and Immunology, Utrecht University, Utrecht, The Netherlands; 2 Department of Immunology, Microbiology and Parasitology, Faculty of Pharmacy, University of the Basque Country (UPV-EHU), Vitoria-Gasteiz, Spain; 3 Produce Safety and Microbiology Research Unit, Agricultural Research Service, US Department of Agriculture, Albany, California, United States of America; East Carolina University School of Medicine, United States of America

## Abstract

The extracytoplasmic function (ECF) σ factors are fundamental for bacterial adaptation to distinct environments and for survival under different stress conditions. The emerging pathogen *Arcobacter butzleri* possesses seven putative pairs of σ/anti-σ factors belonging to the ECF family. Here, we report the identification of the genes regulated by five out of the seven *A. butzleri* ECF σ factors. Three of the ECF σ factors play an apparent role in transport, energy generation and the maintenance of redox balance. Several genes like the *nap*, *sox* and *tct* genes are regulated by more than one ECF σ factor, indicating that the *A. butzleri* ECF σ factors form a network of overlapping regulons. In contrast to other eubacteria, these *A. butzleri* ECF regulons appear to primarily regulate responses to changing environments in order to meet metabolic needs instead of an obvious role in stress adaptation.

## Introduction

*Arcobacter* spp. are Gram-negative, small, motile and spiral-shaped bacteria belonging to the *Campylobacteraceae* family. *Arcobacter* is currently comprised of fifteen species [1], [2]. Within this genus, *A. butzleri*, *A. cryaerophilus* and *A. skirrowii* are associated with animal and human diseases, such as reproductive disorders, mastitis and diarrhoea in animals [3]–[6], and enteritis and occasionally bacteraemia in humans [7]–[10]. *Arcobacter* spp. have been classified in 2002 as emerging pathogens by the ICMSF (International Commission on Microbiological Specifications for Foods) [11].The main reservoirs for *Arcobacter* spp. are water environments, especially sewage and coastal waters [12]–[14].

In 2007 the complete genome of the human clinical isolate *A. butzleri* RM4018 was sequenced [15]. The bacterium appears to have a large number of signal transduction systems, indicating that this organism is able to respond to many different environmental signals. Apart from having one of the highest densities of two component systems per megabase, *A. butzleri* RM4018, unlike *Campylobacter* spp. or *Helicobacter* spp., is also predicted to contain seven σ/anti-σ factor pairs belonging to the ECF family (Extracytoplasmic function) of sigma factors. The seven *A. butzleri* RM4018 ECF σ factor pairs are genetically unlinked but for each pair, the σ factor gene is positioned adjacent to and in the same orientation as the putative cognate anti-σ factor gene. The amino acid identity between any two of the seven ECF σ factors varies between 30 and 49%, suggesting that each ECF pair plays a different role in the biology of strain RM4018 and responds perhaps to a different suite of external signals.

ECF σ factors have been identified in both Gram-negative and Gram-positive bacteria and they are fundamental for bacterial adaptation to different environments and for survival under different stress conditions [16], [17]. Their role in cellular physiology is highly variable and includes adaptation to membrane-affecting compounds, extreme temperatures and pH, light, high pressure, carbon, nitrogen and iron starvation, oxidative and osmotic stress, and the regulation of virulence in pathogenic organisms [16], [18]. Despite their diverse effects, ECF σ factors share common features. In most cases ECF σ factors are co-transcribed with their cognate anti-σ factor. The anti-σ factor, which often spans the inner membrane, sequesters and inactivates the cognate σ factor at the inner face of the cytoplasmic membrane. Upon a specific external stimulus, the ECF anti-σ factor is inactivated by protein degradation and the σ factor is released from the membrane, where it associates with RNA polymerase and mediates transcriptional activation of its target genes [19]. The ECF anti-σ factor can also be released from the ECF σ factor by a phosphorylated response regulator NepR, this partner-switching mechanism has recently be discovered in alphaproteobacteria [20]. Some ECF σ/anti-σ factors form together with an outer membrane TonB-dependent receptor, a trans-envelope signal transduction pathway [31]. These TonB-dependent receptors are involved in the uptake of specific molecules but also they sense and transmit, via the anti-σ factor, extracellular signals, which lead to the activation of a specific ECF σ factor.

In the present study, we investigated the role of the *A. butzleri* RM4018 ECF σ factors. We developed the genetic tools to manipulate *A*. *butzleri* and determined the regulons of most ECF σ factors using a combination of microarray-based transcriptome analysis and functional assays.

## Materials and Methods

### Bacterial Strains, Plasmids and Growth Conditions

Bacterial strains and plasmids used in this work are listed in Table 1. *A. butzleri* strains were routinely grown at 30°C in Brain Heart Infusion (BHI) broth (Oxoid) or on Mueller Hinton (MH) agar (Oxoid) supplemented with 5% sheep blood (Biotrading). *E. coli* strains were routinely grown at 37°C in Luria-Bertani (LB) broth or on LB agar plates (Biotrading) supplemented with ampicillin (100 µg/ml) or kanamycin (50 µg/ml) when needed.

**Table 1:** Bacterial strains and plasmids used in this study.

Bacterial strain or plasmid	Origin/function	Source
***A. butzleri*** ** strains**		
*A. butzleri* RM4018	Human clinicalisolate (ATCC 49616)	USDA*
*A. butzleri* Δσ^1^/Aσ^1^::Km	RM4018 derivative Δ*ab0983–0984*::*aph(3′)-III*	This study
*A. butzleri* ΔAσ^1^::Km	RM4018 derivative Δ*ab0984*::*aph(3′)-III*	This study
*A. butzleri* Δσ^2^/Aσ^2^::Km	RM4018 derivative Δ*ab1040–1041*::*aph(3′)-III*	This study
*A. butzleri* ΔAσ^2^::Km	RM4018 derivative Δ*ab1041*::*aph(3′)-III*	This study
*A. butzleri* Δσ^4^/Aσ^4^::Km	RM4018 derivative Δ*ab1452–1453*::*aph(3′)-III*	This study
*A. butzleri* ΔAσ4::Km	RM4018 derivative Δ*ab1453*::*aph(3′)-III*	This study
*A. butzleri* Δσ^5^/Aσ^5^::Km	RM4018 derivative Δ*ab1567–1568*::*aph(3′)-III*	This study
*A. butzleri* ΔAσ^5^::Km	RM4018 derivative Δ*ab1567*::*aph(3′)-III*	This study
*A. butzleri* Δσ^7^/Aσ^7^::Km	RM4018 derivative Δ*ab2315–2316*::*aph(3′)-III*	This study
*A. butzleri* Δσ^7^::Km	RM4018 derivative Δ*ab2315*::*aph(3′)-III*	This study
***E. coli*** ** strains**		
*E. coli* DH5α NCCB2955	Competent cells for cloning	NCCB**
**Plasmids**		
pGEM-T Easy	Cloning vector, Amp^r^	Promega
pGEMab0983–0984	pGEM-T Easy containing *ab0983–0984*	This study
pGEMab1040–1041	pGEM-T Easy containing *ab1040–1041*	This study
pGEMab1429–1430	pGEM-T Easy containing *ab1429–1430*	This study
pGEMab1452–1453	pGEM-T Easy containing *ab1452–1453*	This study
pGEMab1567–1568	pGEM-T Easy containing *ab1567–1568*	This study
pGEMab2164–2165	pGEM-T Easy containing *ab2164–2165*	This study
pGEMab2315–2316	pGEM-T Easy containing *ab2315–2316*	This study
pGEMΔab0983–0984	pGEM-T Easy containing Δ*ab0983–0984*	This study
pGEMΔab1040–1041	pGEM-T Easy containing Δ*ab1040–1041*	This study
pGEMΔab1429–1430	pGEM-T Easy containing Δ*ab1429–1430*	This study
pGEMΔab1452–1453	pGEM-T Easy containing Δ*ab1452–1453*	This study
pGEMΔab1567–1568	pGEM-T Easy containing Δ*ab1567–1568*	This study
pGEMΔab2164–2165	pGEM-T Easy containing Δ*ab2164–2165*	This study
pGEMΔab2315–2316	pGEM-T Easy containing Δ*ab2315–2316*	This study
pGEMΔab0984	pGEM-T Easy containing Δ*ab0984*	This study
pGEMΔab1041	pGEM-T Easy containing Δ*ab1041*	This study
pGEMΔab1429	pGEM-T Easy containing Δ*ab1429*	This study
pGEMΔab1453	pGEM-T Easy containing Δ*ab1453*	This study
pGEMΔab1568	pGEM-T Easy containing Δ*ab1568*	This study
pGEMΔab2164	pGEM-T Easy containing Δ*ab2164*	This study
pGEMΔab2316	pGEM-T Easy containing Δ*ab2316*	This study
pGEMΔab1040–1041::km	pGEM-T Easy containing Δ*ab1040–1041*::*aph(3′)-III*	This study
pGEMΔab1429–1430::km	pGEM-T Easy containing Δ*ab1429–1430*::*aph(3′)-III*	This study
pGEMΔab1452–1453::km	pGEM-T Easy containing Δ*ab1452–1453*::*aph(3′)-III*	This study
pGEMΔab1567–1568::km	pGEM-T Easy containing Δ*ab1567–1568*::*aph(3′)-III*	This study
pGEMΔab2164–2165::km	pGEM-T Easy containing Δ*ab2164–2165*::*aph(3′)-III*	This study
pGEMΔab2315–2316::km	pGEM-T Easy containing Δ*ab2315–2316*::*aph(3′)-III*	This study
pGEMΔab0984::km	pGEM-T Easy containing Δ*ab0984*::*aph(3′)-III*	This study
pGEMΔab1041::km	pGEM-T Easy containing Δ*ab1041*::*aph(3′)-III*	This study
pGEMΔab1429::km	pGEM-T Easy containing Δ*ab1429*::*aph(3′)-III*	This study
pGEMΔab1453::km	pGEM-T Easy containing Δ*ab1453*::*aph(3′)-III*	This study
pGEMΔab1568::km	pGEM-T Easy containing Δ*ab1568*::*aph(3′)-III*	This study
pGEMΔab2164::km	pGEM-T Easy containing Δ*ab2164*::*aph(3′)-III*	This study
pGEMΔab2316::km	pGEM-T Easy containing Δ*ab2316*::*aph(3′)-III*	This study
pMW2	Plasmid with kanamycin resistance (*aph(3′)-III*) gene from *Campylobacter.*	[21]

* USDA, Agricultural Research Service, California, USA.

** The Netherlands Culture Collection of Bacteria.

### Construction of A. Butzleri ECF σ Factor and ECF Anti-σ Factor Mutants

The genes encoding the seven ECF σ factors and their cognate anti-σ factors were amplified using the primer pairs AB0986F/AB0987R, AB1044F/AB1044R, AB1437F2/AB1437R2, AB1460F/AB1460R, AB1576F/1576R, AB2151F/AB2151R and AB2300F/2300R (Table 2), the proofreading enzyme *Pfu* (Promega) and *A. butzleri* RM4018 chromosomal DNA as template. The PCR products were tailed with a 5′-A nucleotide using *Taq* polymerase (Invitrogen) and ligated into the pGEM-T Easy vector (Promega) to obtain pGEMab0983–0984, pGEMab1040–1041, pGEMab1429–1430, pGEMab1452–1453, pGEMab1567–1568, pGEMab2164–2165 and pGEMab2315–2316 (Table 1). Inverse PCR was performed on the σ/anti-σ plasmids to delete a large part of both the σ and anti-σ factor (σ/anti-σ knock-out plasmids) or of only the anti-σ factor encoding genes (anti-σ knock-out plasmids). Unique *Bam*HI restriction sites were introduced at the same time. The plasmid pGEMΔab1429–1430 was obtained by digesting plasmid pGEMΔab1429 with *Bam*HI and *Bcl*I. The knockout constructs were created by digestion of the inverse PCR products with *Bam*HI and ligation to a 1.4-kb *Bam*HI fragment containing a kanamycin resistance gene (*aph(3′)-III*) from pMW2 [21].

**Table 2:** Oligonucleotides used in this study.

Primer name	DNA sequence (5′ → 3′)
**Mutant construction**	
AB0986F	AACAGTTGCTGATATTAAAGCTATG
AB0986R	TCTTTTTATACCATCAAATGCTGC
AB0986F-bamHI	AGGATCCTGATATTTGAAGATGAAAAATCTTCTA
AB0986R-bamHI	AGGATCCAGTGTTCATTTTCTTCAAAAGG
Arcoantiσ1-bamHI	AGGATCCGTTCAAAAGAGGTTAAACCTTC
AB1044F	TAAGTTTAGTTTCAAAAATCTCTGA
AB1044R	AACACTATCTCCAAATTTGATATC
AB1044F-bamHI	TGGATCCGAAAAAGTATCTAAAACGGAA
AB1044R-bamHI	TGGATCCTATTGGTGAGATTTTATCTATGT
Arcoantiσ2-bamHI	AGGATCCCTAACCACTCTTTTAATTCAATT
AB1437F2	CTTATGATTTTTTATAAATATATATGTA
AB1437R2	ATGCTTAAATATTATGATGAGTT
AB1437R-bamHI	AGGATCCCTATTCTAACATCTGGTAAATG
Arcoantiσ3-bamHI	AGGATCCTACATGTAAAAAAATAAGCTGC
AB1460F	ACTATTCCAATTATTACAGCATT
AB1460R	AGAAAATCCTCTTATTACATTGTAA
AB1460F-bamHI	AGGATCCAGTGGGAATTTTGAAGTC
AB1460R-bamHI	CGGATCCTTGTGCATTATCTTTGTTTGG
Arcoantiσ4-bamHI	AGGATCCTGGTAAGGCTTTTATTTGAGTT
AB1576F	TGCTGTAAAGTTTTACAATAAAGAA
AB1576R	TATTTCCATCTTCAAGAGCTT
AB1576F-bamHI	AGGATCCTGCTCTTTGGTTTTCAATTT
AB1576R-bamHI	TGGATCCGGTGAATTTTCATCAACTCA
Arcoantiσ5-bamHI	AGGATCCATATGTTGCTTCTTCTTTGATT
AB2151F	TTCTAATACTGTTCCTTGACC
AB2151R	TTTTCACAAGAGCATTTATCG
AB2151F-bamHI	TGGATCCTACAAGTTTGGAAACATAG
AB2151R-bamHI	TGGATCCGGAAAAACAGATATAGAAGAGA
Arcoantiσ6-bamHI	TGGATCCTCCACATAAAATAGCAGCCATT
AB2300F	ACAAATCAAGTTTTGCGGCA
AB2300R	CTTAATAAAGCCACTCCTTTG
AB2300F-bamHI	AGGATCCTCATGGGCTAAATCTTTGTC
AB2300R-bamHI	AGGATCCGTTGAACAAATACCAGAAAATA
Arcoantiσ7-bamHI	AGGATCCAACAACTCAACCAATATCTTGCTT
**qRT-PCR**	
arconapBtaqF	TGACCACTTATCAAATGCGAGATT
arconapBtaqR	GGAACATTTGCACTATTTGATTGTG
arconapAtaqF	GCCGCTGCGGCTGTT
arconapAtaqR	CGCCAACCGCCTTCTG
arcosoxDtaqF	TGCGCAATGTGTTATGTGTCA
arcosoxDtaqR	GAACCACCAGCAAGCATAGGA
arcosoxAtaqF	CAGTGCATAGACTAAGATGGGAAGAA
arcosoxAtaqR	GCAACTTGTCCTTGGTCAACAA
Abu100tctAtaqF	GGATTAGCTGCACCCGAGAGT
Abu100tctAtaqR	CGTTCCACTTCCTGGAATTCC
Abu98tctCtaqF	ACAGCAGCAAGACAACAAAATACTTT
Abu98tctCtaqR	AAGATTGAGGAAATACACCTTGTAATGA
AB0988Ftaq	ATAAAAGAGGTAGTGTCGAAAGTGGTT
AB0988Rtaq	CGGTTGATGTGGCTTGTAAATTC
AB1462Ftaq	TTCCAAGAAATCCAAATGAACATG
AB1462Rtaq	AAGCACTCCATTGGTCATTAAATACTATAT
AB1573Ftaq	ACCATAACTTATACTAGCATTCCCTTTTG
AB1473Rtaq	GTACGTGGAGCAACAGGACTAACTAC
arcogyrAtaqF2	AAAGTTTCTCTTTAAGAGCTCCACTTG
arcogyrAtaqR2	TGTATATCTCATAGCCGCTGCATT

*Bam*HI restriction sites are underlined.

The ECF σ factor genes were inactivated by marker exchange mutagenesis. First, the knock-out plasmids were introduced into *A. butzleri* RM4018 by electrotransformation. To obtain electrocompetent *A. butzleri* RM4018, a 5 ml overnight culture was diluted 20 times in 100 ml fresh BHI medium and incubated at 30°C on a shaking platform (150 rpm). Bacteria were harvested by centrifugation (4,500×g, 1 h, 4°C) when the optical density at 550 nm had reached values between 0.2 and 0.6. The bacteria were washed twice in 5 ml of ice-cold sucrose-glycerol solution (15% glycerol; 272 mM sucrose in water), resuspended in 0.5 ml ice-cold sucrose-glycerol solution and aliquoted into 50 µl solutions containing approximately 3−5×10^9^ CFU. One µg of each plasmid was added to 50 µl (approximately 3−5×10^9^ CFU) of competent *A. butzleri* RM4018 and incubated for 3 min on ice. The cells were transferred to a 0.2 cm electroporation cuvette (Bio-Rad) and electroporated using a Bio-Rad Gene Pulser set at 2.25 kV, 400 Ω and 25 µF. Bacteria were recovered in 1 ml of BHI broth for 10 min at room temperature, transferred to 2 ml of pre-warmed BHI and incubated for 3 hours at 30°C on a shaking platform (150 rpm). Mutants were selected by growing the cells for two to five days at 30°C aerobically on kanamycin-containing MH plates. Homologous recombination resulting in double-crossover events was verified by PCR.

### Phenotype Characterization

Growth curves were generated by diluting pre-cultures grown overnight in BHI to a starting optical density (OD_550_) of 0.05 in 30 ml of BHI. The 30 ml cultures were grown in conical flask under aerobic conditions, 150 rpm at 30°C. Bacterial growth and cell density were monitored by measuring the absorbance at 550 nm at different time intervals. The exponential growth rate was calculated from four separated growth experiments. Several stress conditions which limited the growth of the wildtype *Arcobacter* strain were tested. To measure the influence of extreme temperatures, the starting cultures were incubated at 4°C or 60°C for 15 min prior to their incubation at 30°C. To study the effect of the pH on the growth of the strains, the pH of the BHI was adjusted to pH 5 or 9. Osmotic stress was tested by adding 0.35 M NaCl to the BHI. In additional experiments, other stress-inducing chemicals were added to the BHI to concentrations which limited the growth of the wildtype *Arcobacter* strain. These chemicals included: ethanol (5%); SDS (1%); the iron chelator 2,2-dipyridyl (300 µM); the oxidative stress-inducing chemicals H_2_O_2_ (0.04%) and diamide (2 mM); or antimicrobial compounds such as penicillin G and polymyxin B (25 µg/ml). Motility assays were performed by stabbing the strains with a pipette tip into semisolid medium (thioglycolate medium containing 0.4% agar, Difco), followed by incubations under aerobic conditions at 30°C or 37°C for 48 hours.

### RNA Isolation

Overnight grown cultures of *A. butzleri* were diluted to an OD_550_ of 0.1 in BHI and incubated at 30°C on a shaking platform set at 150 rpm. RNA was isolated from 5 ml of mid-logarithmic phase cultures (OD_550_ of approximately 0.5), using the RNA-Bee™ kit (Tel-Test, Inc) following the manufactureŕs specifications.

### Microarray Hybridization and Analysis

For expression profiling, an indirect comparison of gene expression levels was performed [22], [23]. In this microarray experimental design, each labeled cDNA was combined with labeled genomic DNA from *A. butzleri* RM4018. Mixtures were hybridized to a previously designed and manufactured *A. butzleri* DNA array [15]. Labeling of RNA and DNA, hybridization procedure and microarray data analysis were performed as previously described [24]. Details of the microarray have been deposited in the NCBI GEO repository (http://www.ncbi.nlm.nih.gov/geo/) under platform accession number GPL14948. The microarray data set has been deposited in the NCBI Gene Expression Omnibus (GEO) repository (http://www.ncbi.nlm.nih.gov/geo/) under accession number GSE34089.

### Real-time RT-PCR

Primers used in this assay were designed using the Primer Express software (Applied Biosystems) and are listed in Table 2. Prior to amplification, RNA samples were treated with RNase-free DNase I (Invitrogen). RT-PCR was performed on 0.2 µg of DNase I treated RNA with 1 µM of primers and the SYBR® Green I kit (Eurogentec) using a LightCycler® 480 Real-Time PCR System (Roche). The PCR parameters were 30 min at 48°C; 10 min at 95°C, 40 cycles of 95°C for 15 s and 60°C for 1 min and a final dissociation step of 95°C for 15 s, 60°C for 20 s and 95°C for 15 s. To compensate for the variance in the amount of mRNA in the reactions, the calculated threshold cycle (*C*_t_) for each gene amplification was normalized to the *C*_t_ values for the *gyrA* gene. Fold changes were calculated according to the ΔΔ*C*_t_ method [25]. Each sample was examined in four replicates and was repeated with at least two independent preparations of RNA. Standard deviations were calculated and displayed as error bars.

### Nitrate/nitrite Assay

The nitrate reductase activity was determined by measuring the production of nitrite from nitrate as previously described [26]. Briefly, strains were grown overnight (16 h) under aerobic conditions in BHI broth containing 20 mM of sodium nitrate. Nitrite accumulation in the supernatant was detected by mixing 50 µl of the culture supernatants with 850 µl of 1% (w/v) sulphanilamide dissolved in 1 M of HCl and 100 µl of 0.02% (w/v) naphthylethylenediamine solution. After 15 minutes the formation of p-sulfobenzene-azo α-naphthylamine was measured at 540 nm. The amount of nitrite present in 50 µl culture supernatant was estimated using a nitrite standard curve. The nitrite production was adjusted to the total bacterial proteins present in the culture as estimated by using the BCA protein assay kit. To determine the nitrate concentration in *Arcobacter* culture supernatants the supernatants were 10 times diluted in BHI. The diluted culture supernatants were incubated with sulphanilamide and naphthylethylenediamine as described for the nitrite detection. Next a trace amount of zinc dust was added to the mixture. The zinc dust reduces nitrate to nitrite and a red color develops due to the formation of p-sulfobenzene-azo α-naphthylamine. After 2 minutes the zinc has settled on the bottom and 900 µl of the supernatant was transferred to a new tube and the absorbance of the red color was measured at 540 nm. The amount of nitrate present in 50 µl culture supernatant was estimated using a nitrate standard curve which was also treated with zinc dust. Reactions were measured four times and repeated with two independent cultures.

### Sox Enzyme Activity Assay

Overnight cultures in BHI were washed once with *Arcobacter* Minimal 1 (AM1) medium (4.2 mM Na_2_HPO_4_, 2.4 mM KH_2_PO_4_, 9 mM NaCL, 19 mM NH_4_Cl 1×M9 salts, 1 mM of MgCl_2_, 0.2% of sodium pyruvate, 0.02% of FeNH_4_citrate; adjusted to pH 7.5) amended with 20 mM of sodium sulfite, and 10 µg of phenol red per milliliter. The bacteria were subsequently diluted in sodium sulfite-, phenol red-amended AM1 medium to an OD_550_ of 0.05 and allowed to grow in completely filled capped 5 cm glass vials. The strains which were able to oxidize sulfur caused a drop in the pH that was visible as a color change from red to yellow. After 6 hour of growth at 30°C, absorbance at 564 nm was measured and the results obtained for the wild-type strain were set at 100%.

### Citrate Uptake Assay

The ability of the strains to utilize citrate was determined using Simmons citrate agar (Oxoid). Strains grown overnight on MH agar supplemented with 5% sheep blood were streaked onto Simmons Citrate Agar supplemented with 0.01% sodium pyruvate. Cells were incubated at 30°C under aerobic conditions for 48 hours. The strains which were able to use the citrate as carbon source increased the pH of medium, resulting in a color change of the medium from green to blue.

### Statistical Analyses

Analysis of variance (ANOVA) was used to identify if any of the mutations significantly changed the growth rate of the *Arcobacter* strains. Statistical analyses were performed using the SPSS 19.0 statistical package program (SPSS Inc., Chicago, IL).

## Results

### Mutagenesis of the ECF σ Factor and Anti-σ Factor Encoding Genes

For each ECF σ/anti-σ pair, mutational inactivation of the anti-σ factor results in constitutive induction of the σ response, while mutational inactivation of the σ factor results in the down-regulation of ECF-dependent transcription [19], [27]. This allowed us to study the function of the ECFs in *A. butzleri* strain RM4018 without knowing the specific signal(s) that activate them. In order to address the role of the seven ECF σ factors in *A. butzleri* RM4018, we inactivated either both the σ and the anti-σ factor, or the anti-σ factor alone. Since no suitable *Arcobacter* antibiotic resistance cassettes were available and the previously-used chloramphenicol cassette of *Campylobacter coli* could not be used since *A. butzleri* RM4018 is resistant to chloramphenicol, we replaced a large part of the ECF coding regions with a kanamycin resistance gene (*aph(3′)-III*) of *C. coli* [21]. After modifying the *Arcobacter* mutagenesis protocol described by Ho *et al*. [28], we were able to replace the ECF σ/anti-σfactor genes *AB0986/0987* (σ^1^, Aσ^1^), *AB1044/1045* (σ^2^, Aσ^2^), *AB1460/1461* (σ^4^, Aσ^4^), *AB1576/1577* (Aσ^5^, σ^5^) and *AB2300/2301* (Aσ^7^, σ^7^) with the Km^r^ cassette. We also obtained single mutants in the anti-σ factor genes Aσ^1^, Aσ^2^, Aσ^4^, Aσ^5^ and Aσ^7^. All mutants contained the Km^r^ cassette in the same orientation as the ECF genes. As no suitable *Arcobacter* shuttle plasmids exist and no other antibiotic cassettes could be used, we were unable to perform complementation studies.

### Phenotype Characterization

**Figure 1:**
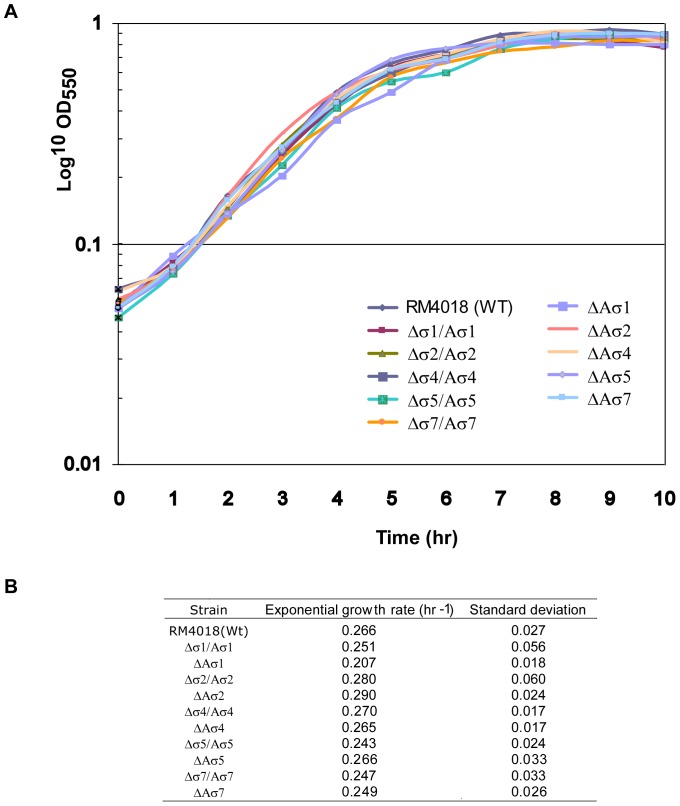
*Arcobacter* growth curves and rates. (A) Growth curves of the wild-type strain RM4018, the ECF anti-σ mutants and the ECF σ/anti-σ double mutants. Strains were grown in BHI at 30°C under aerobic conditions. Data correspond to one representative of four independent experiments. (B) Exponential growth rates (hr^−1^) with standard deviations of four growth curves including one shown in (A).

Comparison of the various mutants with the parent strain RM4018 revealed neither differences in bacterial shape or colony formation on sheep blood plates nor in growth rate in BHI medium as statistically estimated by Anova F = 1,777 (P>0.05) (Figure 1A and B). A common role of ECF σ factors is to protect bacteria against external stress [16]. Exposure of the *A. butzleri* mutant strains to extreme temperatures (e.g., 4°C or 60°C), pH 5 or 9, or sub-inhibitory concentrations of stress-inducing chemicals (e.g., ethanol, SDS, metal ion chelators EDTA or 2,2-dipyridyl, the oxidative stress-inducing chemicals H_2_O_2_ and diamide, or the antimicrobial compounds penicillin G and polymyxin B) did not yield significant growth or morphology differences (data not shown). Similarly, the motility of the mutants on semisolid medium was unchanged compared to the wild-type. These results indicated that the functions of the *Arcobacter* ECF σ factors might be different from those in other bacterial species.

### Identification of ECFσ Factor-regulated Genes

To identify the genes regulated by the RM4018 ECF σ factors, RNA was isolated from wild-type and mutant strains grown to mid logarithmic phase and subjected to microarray-based transcriptome analysis. To obtain maximal ECF σ factor-dependent transcript differences, transcripts of each σ/Aσ mutant and its cognate Aσ mutant were compared. Genes showing more than a fourfold change in transcript levels were considered as ECF σ-dependent. Based on this criterion, the ECF σ factors σ^1^, σ^2^, σ^4^, σ^5^ and σ^7^ were found to regulate 3, 65, 14, 42 and 72 genes, respectively (Tables S1 to S5). None of the ECF σ factors appeared to regulate its own transcription. Interestingly, more than thirty genes were regulated by two or more ECF σ factors (Table 3). In agreement with the phenotypic characterization, the identified ECF σ-dependent genes indicate that the ECF σ factors of *A. butzleri* regulate other genes than ECF σ factors in other bacterial species.

**Table 3:** Genes regulated by two or more ECF σ factors.

Functional class^a^	ORF^a^	Gene^a^	Transcription activated by	Transcription repressed by
Energymetabolism: anaerobic respiration	AB0345	*nrfA*	σ^2^ and σ^4^	
	AB0354	*napH*	σ^2^ and σ^4^	
	AB0355	*napG*	σ^2^ and σ^4^	
	AB0356	*napA*	σ^2^ and σ^4^	
	AB0297	*frdA*	σ^4^ and σ^5^	
Energymetabolism: aerobic respiration.	AB1442	*hyaB*	σ^2^ and σ^4^	σ^7^
Energymetabolism: pyruvate dehydrogenase.	AB1481	*aceF*	σ^4^ and σ^5^	
Energymetabolism: electrontransport.	AB2055	*petA*	σ^5^	
Transport/binding proteins: carbohydrates, organicacids and alcohols	AB0357	*dctP*	σ^2^ and σ^7^	σ^7^
	AB0358	*dctQ*	σ^2^	σ^7^
	AB0359	*dctM*	σ^2^	σ^7^
	AB0102		σ^5^ and σ^7^	σ^2^
	AB0103		σ^5^ and σ^7^	σ^2^
	AB0104		σ^5^ and σ^7^	σ^2^
Transport/binding proteins: others	AB0504		σ^2^and σ^5^	σ^7^
Central intermediary metabolism: general	AB0376	*ald*	σ^2^	σ^7^
	AB0107	*cynT1*	σ^5^	σ^7^
Central intermediary metabolism: sulfur metabolism.	AB0563	*soxC*	σ^2^	σ^7^
	AB0564	*soxD*	σ^2^	σ^7^
	AB0565	*soxX*	σ^2^	σ^7^
	AB0566	*soxY*	σ^2^	σ^7^
	AB0567	*soxZ*	σ^2^	σ^7^
	AB0568	*soxA*	σ^2^	σ^7^
	AB0569		σ^2^	σ^7^
	AB0570	*soxB*	σ^2^	σ^7^
Small molecule metabolism:carbon compound degradation.	AB0494	*ackA1*	σ^2^	σ^7^
	AB0495	*pta*	σ^2^	σ^7^
Cell processes: detoxification.	AB1553	*katG*	σ^2^	σ^7^
Signal transduction: two-component system.	AB0105		σ^7^	σ^2^
	AB0106		σ^7^	σ^2^

a The functions of the encoded proteins and the AB numbers are indicated according to Miller et al. [15].

### Genes Regulated by AB0986 (ECF σ^1^)

The transcriptome analysis revealed that σ^1^ activates the transcription of *AB0988*, encoding a putative TonB-dependent receptor protein, and down-regulates the transcription of *AB1593* and *AB0033*, that encode a putative sodium: alanine symporter and a L-lactate permease, respectively (Table S1).

### Genes Regulated by AB1044 (ECF σ^2^)

Based on the microarray data, ECF σ^2^ activates the transcription of 35 genes and caused a down-regulation of the expression of 30 genes (Table S2). All annotated gene products represent putative proteins. The sodium: solute symporter AB0504 is the most strongly up-regulated gene product. Apart from this, ECF σ^2^ is predicted to control the transcription of the genetic loci: *AB0345*-*AB0346*, encoding a Nrf-type nitrite reductase; *AB0353* to *AB0359*, coding for a Nap- type nitrate reductase and a C_4_-dicarboxylate transport system; *AB0376*–*0377*, encoding an aldehyde dehydrogenase and an uncharacterized protein; *AB0494*–*AB0495*, encoding an acetate kinase and a phosphate acetyltransferase; *AB0563* to *AB0570*, encoding the Sox multi-enzyme complex; and *AB1442*–*1443*, encoding a Ni/Fe-hydrogenase. A putative operon (*AB0102*–*0106*) coding for the tricarboxylic transport proteins TctABC and a large locus (*AB1664* to *AB1682*) coding for phage related proteins were among the genes that are down-regulated by ECF σ^2^.

### Genes Regulated by AB1460 (ECF σ^4^)

According to the transcriptomics data, σ^4^ activates 11 genes and caused a down-regulation of the expression of 3 genes (Table S3). The main up-regulated gene product is the putative TonB-dependent receptor protein (AB1462) encoded by the same locus encoding the σ^4^/anti-σ^4^ factors. Among the other genes up-regulated by ECF σ^4^ are: *napHGA* (*AB0354* to *AB0356*), *aceEF* (*AB1480*–*1481*), *frdA* (*AB0297*), *nrfA (AB0345*) and *fdhA1* (*AB1507*) encoding, respectively, a part of a Nap-type nitrate reductase, two pyruvate dehydrogenase components, a fumarate reductase subunit, a cytochrome c_552_ nitrate reductase and a formate dehydrogenase subunit. A methyl-accepting chemotaxis protein (AB0602) is the most strongly down-regulated gene product.

### Genes Regulated by AB1577 (ECF σ^5^)

Identification of ECF σ^5^-dependent genes by transcriptomics analysis revealed 16 up-regulated and 26 down-regulated genes (Table S4). The closely-linked TonB-dependent receptor gene *AB1573*, was the most strongly up-regulated. The putative TctABC transport proteins AB0102–AB0104, the carbonic anhydrase AB0107, the putative ammonia monooxygenase AB0108, and the ubiquinolcytochrome c oxidoreductase encoded by the *petAB* (*AB2054*–*AB2055*) genes are among the gene products up-regulated by σ^5^. The genes *fur2* and *cspA*, and the genetic locus *AB1062*–*1066*, which includes NADPH quinonereductase, NADPH:flavodoxinoxidoreductase, and NADPH nitroreductase, were found to be repressed.

### Genes Regulated by AB2301 (ECF σ^7^)

The transcriptome analysis revealed that σ^7^ activates 37 genes and represses another 35 (Table S5). Almost all annotated genes repressed by σ^7^ were activated by σ^2^. The genes encoding the putative TctABC transport proteins (*AB0103*–*0105*) are the most up-regulated genes by σ^7^, while the *sox* operon and *AB0494*–*AB0495*, encoding an acetate kinase and a phosphate acetyltransferase are the most down-regulated. Although it does not affect the transcription of its linked TonB-dependent receptor gene (AB2299), σ^7^ activated two other TonB-dependent receptor genes *AB0705* and *AB1870*.

### The *A. butzleri* ECF σ Factors Regulate Parts of the Electron Transport Chain

**Figure 2:**
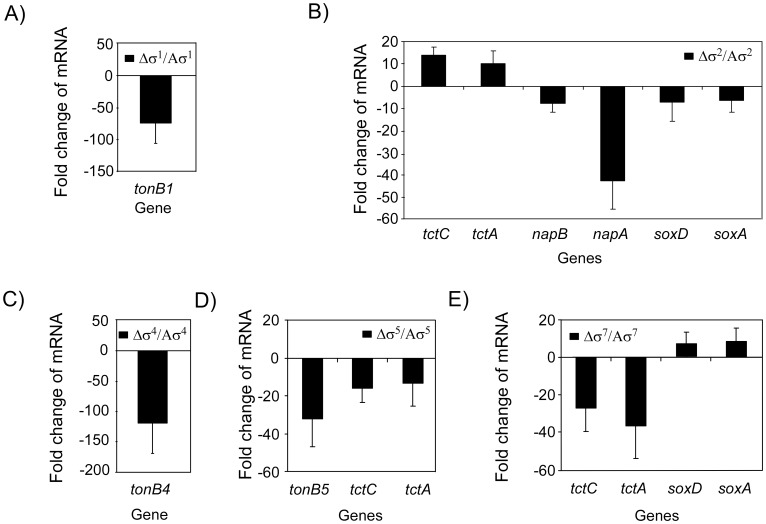
Real-time RT-PCR data confirming parts of the *A. butzleri* ECF regulons. (A) σ^1^, (B) σ^2^, (C) σ^4^, (D) σ^5^, and (E) σ^7^ regulon. Transcripts of each σ/Aσ mutant were compared to the cognate Aσ mutant. Fold change relative to the transcription levels was calculated using the arithmetic formula 2^−ΔΔ*C*t^. Data represent the mean values and standard deviation of four independent experiments with two independent RNA preparations.

Unlike the *tonB* genes which also appear to be regulated by ECF sigma factors in other bacterial species, we found that a large number of genes regulated by *A. butzleri* ECF σ factors are involved in the electron transport chain. To verify that the ECF σ factors indeed influence the electron transport chain, we first performed real-time RT-PCR on the ECF-dependent *tonB*, *nap*, *sox* and *tct* genes. We could confirm that the *tonB* genes *AB0988*, *AB1462* and *AB1573* are indeed up-regulated by ECF σ^1^, σ^4^ and σ^5^ respectively (Figure 2a, c and d). ECF σ^2^ activates the *nap* and *sox* genes (Figure 2b) of which the latter are repressed by σ^7^ (Figure 2e). The real-time RT-PCR results also confirmed that the *tct* genes are down-regulated by ECF σ^2^ and up-regulated by σ^5^ and σ^7^ (Figure 2b, d and e).

**Figure 3:**
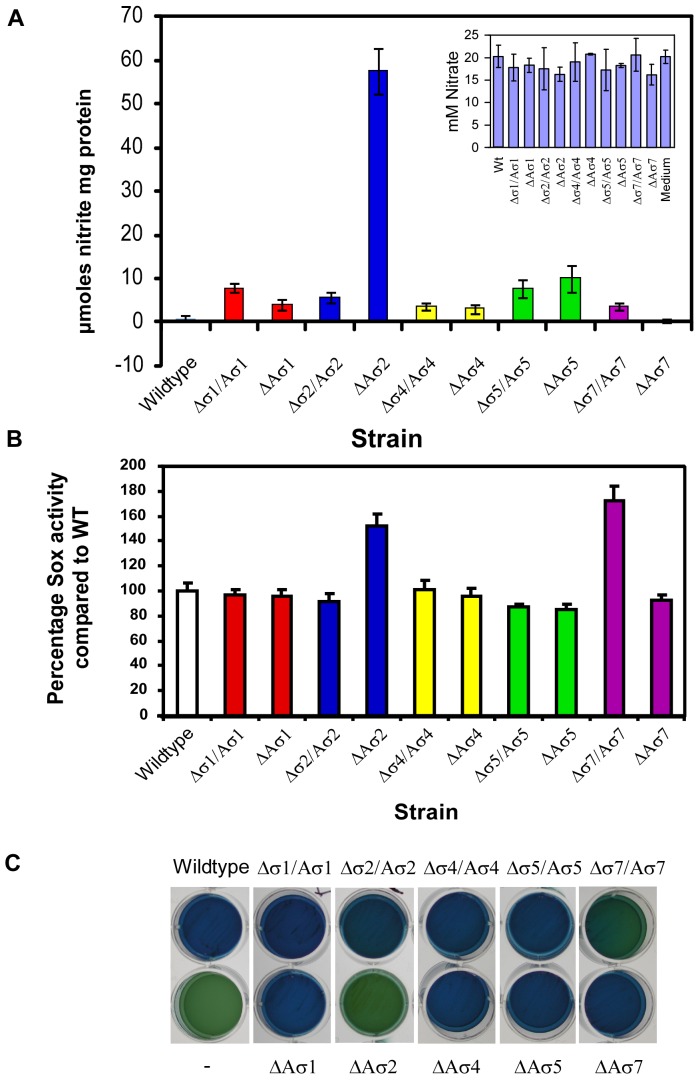
Functional enzyme assays confirming the differences in transcripts between wild-type and ECF mutants. (**A**) Nitrate reductase activity was determined under aerobic conditions. Nitrate and nitrite present in the culture supernatant was measured four times and experiments were repeated with two independent cultures. (**B**) Sox enzyme activity assays performed under anaerobic conditions. Standard errors based on four independent experiments are indicated. (**C**) Utilization of citrate tested on Simmons Citrate Agar. Strains which were able to utilize citrate as carbon source caused a change in pH of the Simmons agar which results in a color change of the medium from green to blue. The sign “−”means no bacteria inoculated.

To prove that the differences in *nap*, *sox* and *tct* transcripts result in phenotypic differences, we determined the nitrate reductase activity, the ability to oxidize sulfate, and the utilization of citrate in ECF mutants compared to the wild-type. As expected, none of the strains except *A. butzleri* Aσ^2^::Km produced nitrite from the supplied nitrate in the presence of oxygen (Figure 3a). The constitutive expression of σ^2^ in the case of Aσ^2^::Km mutant led to nitrite production regardless of the presence of oxygen, indicating that ECF σ^2^ up-regulates the Nap-type nitrate reductase. To investigate whether the altered transcription levels of the *sox* genes resulted in a change in Sox enzyme activity, all the strains were grown for 6 hours in AM1 medium supplemented with sulfite and the pH indicator phenol red. The mutants ΔAσ2 and Δσ7/Aσ7 further induced the change in medium pH observed during growth of the parental strain, indicating that these mutants have an increased ability to oxidize reduced sulfur compounds (Figure 3b). These results confirmed that ECF σ^2^ and σ^7^ regulate the Sox sulfur oxidation proteins in an opposite way. Finally, we investigated whether these strains are able to utilize citrate, which causes an increase in the pH and a color change of the medium when these bacteria are grown on Simmons agar. In agreement with microarray and real-time RT PCR results, all strains except the mutants ΔAσ2 and Δσ7/Aσ7 caused a color change of the media, indicating that the ΔAσ2 and Δσ7/Aσ7mutants could not metabolize citrate (Figure 3c).

## Discussion

Although *Arcobacter* has been classified as an emerging pathogen (ICMSF, 2002), knowledge on this organism is still limited. The genome sequence of *A. butzleri* strain RM4018 [15] revealed that, unlike other characterized members of the epsilon proteobacteria, this strain possesses seven putative extracytoplasmic function (ECF) σ factors. These σ factors are often involved in the regulation of virulence or stress-related genes in other bacteria. In the present study, we developed genetic tools to manipulate *A. butzleri* and used these tools to investigate the function of the putative RM4018 ECF σ factors. *A. butzleri* is resistant to many different antibiotics [15], [29], [30]; therefore, it was difficult to find a suitable antibiotic cassette to inactivate the ECF σ factor-encoding genes. Only a kanamycin resistance gene from *C. coli* [31] appeared to be functional in strain RM4018. By electroporation we were able to generate 10 different mutants and were able to inactivate 5 of the 7 ECF σ factors and to identify the corresponding regulons. Despite repeated attempts, we were unable to mutate the ECF σ factors AB1430 (σ^3^) and AB2165 (σ^6^), which may indicate that these factors are essential for bacterial growth under the conditions employed.

ECF σ factors are involved in a wide range of environmental responses in many bacterial species [18]. They are mainly divided into stress response ECF σ factors and iron-starvation ECF σ factors [32]. Our results indicate that the ECF σ factors of the strain RM4018 are neither involved in iron-starvation nor in the stress response. Furthermore, we found no evidence of positive regulation of their own transcription, as often found for other ECF σ factors [27]. Except for the *tonB* genes, the RM4018 ECF σ factors regulate completely different sets of genes. This seemingly atypical gene regulation in *Arcobacter* has also been noted for the regulation of the flagellar genes as *Arcobacter* lacks the σ factors FliA and RpoN, which in many bacterial species regulate flagellar biosynthesis [15]. So, in contrast to other eubacteria, many conserved genes in *A. butzleri* appear to be regulated by a σ factor belonging to a different σ factor class; therefore, knowledge on transcription regulation of conserved genes described in other bacterial species cannot be simply extrapolated to *A. butzleri*.

In *A. butzleri* RM4018, 5 out the 7 ECF σ/anti σ-factor pairs are flanked by genes encoding putative TonB-receptor proteins. In other species, the ECF σ/anti σ factors form, together with an outer membrane TonB-dependent receptor, a cell-surface signaling (CSS) system [33]. Transcriptome analyses showed that σ^1^, σ^4^ and σ^5^ regulate the expression of the TonB-dependent receptors encoded by the same loci, suggesting the putative presence of three potential trans-envelope signal transduction systems in *A. butzleri* RM4018. Most of the described CSS systems are involved in iron signaling and transport [34]. However, other functions have been described including for the *Ralstonia solanacearum* Prha-PrhIR system, which senses the presence of a plant cell-wall structure and initiates a regulatory cascade that induces the hypersensitive response and the transcription of pathogenicity genes [35]. The role of the three putative CSS systems in *A. butzleri* RM4018 remains to be elucidated, as the growth of mutants in these systems are not affected by iron limitation.

Transcriptome analysis, as well as functional assays, showed that *A. butzleri* ECF σ factors also regulate a number of genes involved in the binding and transport of specific compounds, energy metabolism and sulfur oxidation. We showed that *Arcobacter* does not reduce nitrate under aerobic conditions (Figure 3A), although it does under anaerobic conditions (data not shown). The ECF σ^2^ is involved in this environmental adaptation as the Aσ^2^::Km mutant produces nitrite under aerobic conditions. This may indicate that oxygen stress has an effect on ECF σ^2^ activity. Several genes must be indirectly regulated by the ECF σ factors as they were up-regulated in the ECF σ mutants. Interestingly and also seen in *Bacillus subtilis* [36], a number of these genes are regulated by more than one ECF σ factor, which indicates that they may be needed under different growth conditions. For example, σ^2^ and σ^7^ regulate the *dctPQM* and *sox* genes in an opposite way. The DctPQM proteins form a tripartite ATP-independent periplasmic transporter, which catalyzes the uptake of C_4_-dicarboxylates like malate, fumarate and succinate in many aerobic bacteria [37]. The Sox proteins which are not found in other members of the *Campylobacteraceae* are involved in the oxidation of reduced sulfur compounds in sulfur, photo- and chemo-lithotrophic bacteria [38]. Similarly, we showed that σ^5^ and σ^7^ activate, while σ^2^ caused a down-regulation of the expression of the *tctABC* genes encoding a tricarboxylic transport system. Despite this, only σ^7^ and σ^2^ appeared to have a significant effect on citrate utilization. In many bacterial species a two-component signal transduction system activated by citrate is responsible for the activation of the *tctABC* genes [39]. In *A. butzleri,* the putative TctABC system may also depend on the two-component system (*AB0105*–*0106*) located directly downstream of the putative *tctABC* genes. The expression of *AB0105*–*0106* was not dramatically affected by σ^5^ and this may explain why citrate utilization was distinct between Δσ5/Aσ5 and Δσ7/Aσ7. All together, the genes regulated by the different *A. butzleri* ECF σ factors indicate that the ECF σ factors form a complex network of regulons that have a major role in regulating bacterial metabolism.

In conclusion, we have shown in this initial study that the ECF σ factors of *A. butzleri* control the transcription of a complex network of regulons that contain genes that are not commonly regulated by ECF σ factors family of proteins. These genes are mainly involved in the energy metabolism. In contrast to other eubacteria, many conserved genes in *A. butzleri* appear to be regulated by a σ factor belonging to a different σ factor class.

## Supporting Information

Table S1 **Genes identified by micro-array analyses which are more than fourfold up or down regulated by** ***A. butzleri*** **ECF sigma 1.**
(DOC)

Table S2 **Genes identified by micro-array analyses which are more than fourfold up or down regulated by** ***A. butzleri*** **ECF sigma 2.**
(DOC)

Table S3 **Genes identified by micro-array analyses which are more than fourfold up or down regulated by** ***A. butzleri*** **ECF sigma 4.**
(DOC)

Table S4 **Genes identified by micro-array analyses which are more than fourfold up or down regulated by** ***A. butzleri*** **ECF sigma 5.**
(DOC)

Table S5 Genes identified by micro-array analyses which are more than fourfold up or down regulated by *A. butzleri* ECF sigma 7.
(DOC)
