# Supplementary material for: Regulation of Energy Metabolism by the Extracytoplasmic Function (ECF) σ Factors of Arcobacter butzleri
Source: PLoS One. 2012 Sep 18;7(9):e44796. doi: 10.1371/journal.pone.0044796 (PMC3445524; doi:10.1371/journal.pone.0044796)
Supplement: Table S1 — Genes identified by micro-array analyses which are more than fourfold up or down regulated by A. butzleri ECF sigma 1. (DOC) [file pone.0044796.s001.doc]

| **Table S1.** σ^1^regulon. | | | |
| --- | --- | --- | --- |
| **ORF^a^** | **Gene** | **Predicted function^a^** | **Fold differences** Δ**Aσ vs** Δ**σ/Aσ^b^** |
| **Genes of *A. butzleri* RM4018 with increased expression in *A. butzleri*** Δ**Aσ^1^** | | | |
| AB0988 |  | TonB-dependent receptor protein | 31.2 |
| **Genes of *A. butzleri* RM4018 with decreased expression in  *A. butzleri*** Δ**Aσ^1^** | | | |
| AB0033 | *lctP* | L-lactate permease | 4.6 |
| AB1593 |  | Sodium:alaninesymporter | 13.4 |

**^b^**The fold difference was calculated by comparison of the RNA levels in *A. butzleri* ΔAσ^1^ with those in *A. butzleri* Δσ^1^/Aσ^1^.

**^a^**The functions of the encoded proteins and the AB numbers are indicated according to Miller et al.[15].
