# Supplementary material for: Regulation of Energy Metabolism by the Extracytoplasmic Function (ECF) σ Factors of Arcobacter butzleri
Source: PLoS One. 2012 Sep 18;7(9):e44796. doi: 10.1371/journal.pone.0044796 (PMC3445524; doi:10.1371/journal.pone.0044796)
Supplement: Table S2 — Genes identified by micro-array analyses which are more than fourfold up or down regulated by A. butzleri ECF sigma 2. (DOC) [file pone.0044796.s002.doc]

| **Table S2**. σ^2^regulon. | | | |
| --- | --- | --- | --- |
| **ORF^a^** | **Gene** | **Predicted function^a^** | **Fold differences** Δ**Aσ vs** Δ**σ/Aσ^b^** |
| **Genes of *A. butzleri* RM4018 with increased expression in *A .butzleri*** Δ**Aσ^2^** | | | |
| AB0345 | *nrfA* | Cytochrome c552 nitrite reductase catalytic subunit NrfA | 5.5 |
| AB0346 | *nrfH* | Cytochrome c nitrite reductase, small subunit NrfH | 5.6 |
| AB0353 | *napB* | Periplasmic nitrate reductase, small subunit, cytochrome c-type protein NapB | 5.7 |
| AB0354 | *napH* | Methylamine utilization ferredoxin-type protein NapH | 6.8 |
| AB0355 | *napG* | Fe-S ferredoxin-type protein NapG | 8.0 |
| AB0356 | *napA* | Periplasmic nitrate reductase, large subunit | 10.1 |
| AB0357 | *dctP* | C4-dicarboxylate-binding periplasmic protein | 7.4 |
| AB0358 | *dctQ* | C4-dicarboxylate transport system, permease small subunit | 6.0 |
| AB0359 | *dctM* | C4-dicarboxylate transport protein | 6.0 |
| AB0376 | *ald* | Aldehyde dehydrogenase | 10.8 |
| AB0377 |  | Conserved hypothetical protein (DUF779 domain protein) | 6.4 |
| AB0463 |  | Conserved hypothetical protein | 4.3 |
| AB0494 | *ackA1* | Acetate kinase | 6.8 |
| AB0495 | *pta* | Phosphate acetyltransferase | 4.7 |
| AB0504 |  | Sodium:solute symporter family protein | 18.4 |
| AB0505 |  | Conserved hypothetical protein (DUF485 domain protein) | 13.5 |
| AB0563 | *soxC* | Sulfur oxidation protein SoxCD, sulfur dehydrogenase subunit | 9.1 |
| AB0564 | *soxD* | Sulfur oxidation protein SoxCD, diheme cytochrome c subunit | 11.4 |
| AB0565 | *soxX* | Sulfur oxidation protein SoxXA, monoheme cytochrome c subunit | 12.6 |
| AB0566 | *soxY* | Sulfur oxidation protein SoxYZ, sulfur covalently binding protein | 14.1 |
| AB0567 | *soxZ* | Sulfur oxidation protein SoxYZ, sulfur compound chelating protein | 10.1 |
| AB0568 | *soxA* | Sulfur oxidation protein SoxXA, diheme cytochrome c subunit | 11.3 |
| AB0569 |  | Hypothetical protein | 9.2 |
| AB0570 | *soxB* | Sulfur oxidation protein, sulfate thiol esterase | 8.2 |
| AB0574 |  | Hypothetical protein | 4.6 |
| AB0910 |  | Conserved hypothetical protein | 6.1 |
| AB1417 |  | Conserved hypothetical protein | 5.2 |
| AB1442 | *hyaB* | Ni/Fe-hydrogenase, large subunit | 6.0 |
| AB1443 | *hyaA* | Ni/Fe-hydrogenase, small subunit | 10.3 |
| AB1526 |  | Conserved hypothetical protein | 4.4 |
| AB1529 |  | Hypothetical protein | 4.7 |
| AB1553 | *katG* | Catalase/peroxidase HPI | 7.3 |
| AB1593 |  | Sodium:alanine symporter | 9.1 |
| AB1961 | *flgG2* | Flagellar distal rod protein FlgG | 4.3 |
| AB2127 |  | Cytochrome c551 peroxidase | 6.1 |
| **Genes of *A. butzleri* RM4018 with decreased expression in *A. butzleri*** Δ**Aσ^2^** | | | |
| AB0102 |  | Conserved hypothetical protein, putative tricarboxylic transport protein TctC | 4.3 |
| AB0103 |  | Conserved hypothetical protein, putative tricarboxylic transport protein TctB | 9.7 |
| AB0104 |  | Conserved hypothetical protein, putative tricarboxylic transport protein TctA | 5.4 |
| AB0105 |  | Two-component response regulator | 6.0 |
| AB0106 |  | Two-component sensor histidine kinase | 7.1 |
| AB0729 | *irgA* | Iron-regulated outer membrane virulence protein homolog | 4.7 |
| AB1022 | *rpsD* | 30S ribosomal protein S4 | 4.1 |
| AB1311 |  | Cytochrome c biogenesis protein | 5.9 |
| AB1664 |  | Hypothetical protein | 4.8 |
| AB1665 |  | Phage major tail tube protein | 4.8 |
| AB1666 |  | Phage tail sheath protein | 6.8 |
| AB1668 |  | Tail fiber assembly protein | 7.6 |
| AB1669 |  | Hypothetical protein | 6.2 |
| AB1670 |  | Tail protein I, putative | 6.3 |
| AB1671 |  | Baseplate assembly protein J, putative | 4.9 |
| AB1672 |  | Hypothetical protein, putative baseplate assembly protein W | 7.1 |
| AB1673 |  | Baseplate assembly protein V, putative | 7.0 |
| AB1674 |  | Hypothetical protein | 7.0 |
| AB1675 |  | Hypothetical protein | 8.6 |
| AB1678 |  | Mu-like prophage I protein, putative | 6.9 |
| AB1679 |  | Hypothetical protein | 7.5 |
| AB1680 |  | Conserved hypothetical protein | 5.8 |
| AB1681 |  | Conserved hypothetical protein | 4.4 |
| AB1682 |  | Conserved hypothetical protein | 6.3 |
| AB1839 |  | Conserved hypothetical protein | 4.1 |
| AB2088 | *htrA* | Periplasmic serine protease DO; heat shock protein HtrA | 5.1 |
| AB2233 |  | DegT/DnrJ/EryC1/StrS aminotransferase | 4.1 |
| AB2303 |  | Sulfatase | 7.2 |
| AB2304 |  | PAP2 superfamily protein | 7.0 |
| **^a^**The functions of the encoded proteins and the AB numbers are indicated according to Miller et al.[15].  **^b^**The fold difference was calculated by comparison of the RNA levels in *A. butzleri* Aσ^2^ with those in *A. butzleri* σ^2^/Aσ^2^. | | | |
