# Supplementary material for: Regulation of Energy Metabolism by the Extracytoplasmic Function (ECF) σ Factors of Arcobacter butzleri
Source: PLoS One. 2012 Sep 18;7(9):e44796. doi: 10.1371/journal.pone.0044796 (PMC3445524; doi:10.1371/journal.pone.0044796)
Supplement: Table S3 — Genes identified by micro-array analyses which are more than fourfold up or down regulated by A. butzleri ECF sigma 4. (DOC) [file pone.0044796.s003.doc]

| **Table S3**. σ^4^regulon. | | | |
| --- | --- | --- | --- |
| **ORF^a^** | **Gene** | **Predicted function^a^** | **Fold differences** Δ**Aσ vs** Δ**σ/Aσ^b^** |
| **Genes of *A. butzleri* RM4018 with increased expression in *A .butzleri*** Δ**Aσ^4^** | | | |
| AB0297 | *frdA* | Fumarate reductase, flavoprotein subunit | 4.9 |
| AB0345 | *nrfA* | Cytochrome c552 nitrite reductase catalytic subunit NrfA | 4.7 |
| AB0354 | *napH* | Methylamine utilization ferredoxin-type protein NapH | 4.7 |
| AB0355 | *napG* | Fe-S ferredoxin-type protein NapG | 5.4 |
| AB0356 | *napA* | Periplasmic nitrate reductase, large subunit | 7.4 |
| AB1442 | *hyaB* | Ni/Fe-hydrogenase, large subunit | 4.8 |
| AB1461 |  | σ factor regulatory protein, FecR/PupR family | 5.1 |
| AB1462 |  | TonB-dependent receptor protein | 75.7 |
| AB1480 | *aceE* | Pyruvate dehydrogenase E1 component | 6.4 |
| AB1481 | *aceF* | Dihydrolipoamideacetyltransferase | 4.8 |
| AB1507 | *fdhA1* | Formate dehydrogenase, large subunitFdhA (selenocysteine-containing) | 6.3 |
| **Genes of *A. butzleri* RM4018 with decreased expression in *A. butzleri*** Δ**Aσ^4^** | | | |
| AB0076 | *rpmI* | 50S ribosomal protein L35 | 5.0 |
| AB0601 |  |  | 5.2 |
| AB0602 |  | Methyl-accepting chemotaxis protein | 6.2 |
| **^a^**The functions of the encoded proteins and the AB numbers are indicated according to Miller et al.[15].  **^b^**The fold difference was calculated by comparison of the RNA levels in *A. butzleri* ΔAσ^4^ with those in *A. butzleri* Δσ^4^/Aσ^4^. | | | |
