# Supplementary material for: Regulation of Energy Metabolism by the Extracytoplasmic Function (ECF) σ Factors of Arcobacter butzleri
Source: PLoS One. 2012 Sep 18;7(9):e44796. doi: 10.1371/journal.pone.0044796 (PMC3445524; doi:10.1371/journal.pone.0044796)
Supplement: Table S4 — Genes identified by micro-array analyses which are more than fourfold up or down regulated by A. butzleri ECF sigma 5. (DOC) [file pone.0044796.s004.doc]

| **Table S4.** σ^5^ regulon. | | | |
| --- | --- | --- | --- |
| **ORF^a^** | **Gene** | **Predicted function^a^** | **Fold differences** Δ**Aσ vs** Δ**σ/Aσ^b^** |
| **Genes of *A. butzleri* RM4018 with increased expression in *A. butzleri*** Δ**Aσ^5^** | | | |
| AB0090 | *pstC* | Phosphate ABC transporter, permease protein | 5.5 |
| AB0102 |  | Conserved hypothetical protein, putative tricarboxylic transport protein TctC | 18.0 |
| AB0103 |  | Conserved hypothetical protein, putative tricarboxylic transport protein TctB | 10.0 |
| AB0104 |  | Conserved hypothetical protein, putative tricarboxylic transport protein TctA | 11.5 |
| AB0107 | *cynT1* | Carbonic anhydrase | 7.2 |
| AB0108 |  | Conserved hypothetical protein, putative ammoniamonooxygenase | 4.5 |
| AB0297 | *frdA* | Fumarate reductase, flavoprotein subunit | 6.8 |
| AB0356 | *napA* | Periplasmic nitrate reductase, large subunit | 6.4 |
| AB1351 |  | Hypothetical protein | 4.2 |
| AB1481 | *aceF* | Dihydrolipoamideacetyltransferase | 4.1 |
| AB1573 |  | TonB-dependent receptor protein | 162.6 |
| AB1607 | *atpA* | ATP synthase F1 sector, alpha subunit | 4.8 |
| AB1724 |  | Conserved hypothetical protein | 4.3 |
| AB1980 | *fus* | Translational elongation factor G | 4.6 |
| AB2054 | *petB* | Ubiquinol cytochrome c oxidoreductase, cytochrome b subunit | 6.2 |
| AB2055 | *petA* | Ubiquinol cytochrome c oxidoreductase, 2Fe-2S subunit | 5.7 |
| **Genes of *A. butzleri* RM4018 with decreased expression in *A .butzleri*** Δ**Aσ^5^** | | | |
| AB0005 | *queF* | 7-cyano-7-deazaguanine reductase | 4.4 |
| AB0068 |  | Conserved hypothetical periplasmic protein | 7.2 |
| AB0076 | *rpmI* | 50S ribosomal protein L35 | 4.3 |
| AB0286 | *tsaA* | Alkyl hydroperoxidereductase/ thiolspecific antioxidant | 5.9 |
| AB0389 |  | Conserved hypothetical protein | 6.3 |
| AB0463 |  | Conserved hypothetical protein | 4.9 |
| AB0741 |  | Conserved hypothetical protein | 4.1 |
| AB0777 |  | Conserved hypothetical protein | 7.5 |
| AB0819 |  | Putative transcriptional regulator (MarR family) | 4.5 |
| AB1062 |  | Flavodoxin-like fold domain protein, putative NADPH-quinonereductase | 10.8 |
| AB1063 |  | Conserved hypothetical protein | 24.6 |
| AB1064 |  | NADH:flavinoxidoreductase/NADH oxidase | 11.5 |
| AB1066 |  | NAD(P)H-flavinnitroreductase | 4.0 |
| AB1572 |  | Conserved hypothetical protein | 4.1 |
| AB1585 | *sodB* | Superoxide dismutase | 4.7 |
| AB1589 |  | Conserved hypothetical protein, putative asparaginase | 4.3 |
| AB1593 |  | Sodium:alaninesymporter | 8.2 |
| AB1767 |  | Hypothetical protein | 4.4 |
| AB1780 |  | Conserved hypothetical protein | 4.5 |
| AB1784 | *fur2* | Ferric uptake regulation protein | 4.9 |
| AB1875 | *cspA* | Cold-shock protein, DNA-binding | 6.7 |
| AB1883 | *ahpC* | Alkyl hydroperoxidereductase/ thiolspecific antioxidant | 4.9 |
| AB2021 | *porA* | Major outer membrane protein | 4.0 |
| AB2118 | *nadD* | Nicotinate (nicotinamide) nucleotide adenylyltransferase | 5.3 |
| AB2141 |  | DNA-binding ferritin-like protein (Dps/NapA) | 6.2 |
| **^a^**The functions of the encoded proteins and the AB numbers are indicated according to Miller et al.[15].  **^b^**The fold difference was calculated by comparison of the RNA levels in *A. butzleri* ΔAσ^5^ with those in *A. butzleri* Δσ^5^/Aσ^5^. | | | |
