# Supplementary material for: Regulation of Energy Metabolism by the Extracytoplasmic Function (ECF) σ Factors of Arcobacter butzleri
Source: PLoS One. 2012 Sep 18;7(9):e44796. doi: 10.1371/journal.pone.0044796 (PMC3445524; doi:10.1371/journal.pone.0044796)
Supplement: Table S5 — Genes identified by micro-array analyses which are more than fourfold up or down regulated by A. butzleri ECF sigma 7. (DOC) [file pone.0044796.s005.doc]

| **Table S5.** σ^7^regulon. | | | |
| --- | --- | --- | --- |
| **ORF^a^** | **Gene** | **Predicted function^a^** |  |
| **Genes of *A. butzleri* RM4018 with increased expression in *A .butzleri*** Δ**Aσ^7^** | | | |
| AB0102 |  | Conserved hypothetical protein, putative tricarboxylic transport protein TctC | 25.7 |
| AB0103 |  | Conserved hypothetical protein, putative tricarboxylic transport protein TctB | 97.7 |
| AB0104 |  | Conserved hypothetical protein, putative tricarboxylic transport protein TctA | 38.4 |
| AB0105 |  | Two-component response regulator | 47.1 |
| AB0106 |  | Two-component sensor histidine kinase | 27.8 |
| AB0107 | *cynT1* | Carbonic anhydrase | 13.9 |
| AB0108 |  | Conserved hypothetical protein, putative ammonia monooxygenase | 4.8 |
| AB0705 |  | TonB-dependent receptor protein | 4.2 |
| AB0707 | *exbB2* | Biopolymer transport protein ExbB | 4.6 |
| AB0725 |  | Two-component response regulator | 6.2 |
| AB0729 | *irgA* | Iron-regulated outer membrane virulence protein homolog | 14.1 |
| AB0730 |  | Conserved hypothetical protein, possible IroE protein | 4.9 |
| AB0756 | *rplD* | 50S ribosomal protein L4 | 4.2 |
| AB0757 | *rplW* | 50S ribosomal protein L23 | 6.8 |
| AB0759 | *rpsS* | 30S ribosomal protein S19 | 7.4 |
| AB0760 | *rplV* | 50S ribosomal protein L22 | 7.9 |
| AB0761 | *rpsC* | 30S ribosomal protein S3 | 6.1 |
| AB0762 | *rplP* | 50S ribosomal protein L16 | 5.9 |
| AB0763 | *rpmC* | 50S ribosomal protein L29 | 4.0 |
| AB0764 | *rpsQ* | 30S ribosomal protein S17 | 10.5 |
| AB0765 | *rplN* | 50S ribosomal protein L14 | 8.0 |
| AB0766 | *rplX* | 50S ribosomal protein L24 | 8.4 |
| AB0767 | *rplE* | 50S ribosomal protein L5 | 4.1 |
| AB0768 | *rpsN* | 30S ribosomal protein S14 | 14.7 |
| AB0769 | *rpsH* | 30S ribosomal protein S8 | 4.2 |
| AB0770 | *rplF* | 50S ribosomal protein L6 | 4.2 |
| AB0772 | *rpsE* | 30S ribosomal protein S5 | 4.6 |
| AB0773 | *rplO* | 50S ribosomal protein L15 | 4.1 |
| AB1606 | *atpG* | ATP synthase F1 sector, gamma subunit | 4.8 |
| AB1608 | *atpH* | ATP synthase F1 sector, delta subunit | 6.1 |
| AB1782 |  | Conserved hypothetical protein | 4.4 |
| AB1862 | *feoA* | Ferrousiron transport protein A | 4.1 |
| AB1866 |  | Conserved hypothetical protein | 6.8 |
| AB1867 |  | Conserved hypothetical protein | 4.4 |
| AB1870 |  | TonB-dependent receptor protein | 7.2 |
| AB1890 | *rplJ* | 50S ribosomal protein L10 | 7.4 |
| AB2296 |  | Conserved hypothetical protein | 4.4 |
| **Genes of *A. butzleri* RM4018 with decreased expression in *A. butzleri*** Δ**Aσ^7^** | | | |
| AB0016 |  | Conserved hypothetical protein, putative MFS permease | 6.2 |
| AB0065 |  | Hypothetical protein | 6.8 |
| AB0068 |  | Conserved hypothetical periplasmic protein | 4.6 |
| AB0357 | *dctP* | C4-dicarboxylate-binding periplasmic protein | 11.0 |
| AB0358 | *dctQ* | C4-dicarboxylate transport system, permease small subunit | 8.6 |
| AB0359 | *dctM* | C4-dicarboxylate transport protein | 8.3 |
| AB0376 | *ald* | Aldehyde dehydrogenase | 8.2 |
| AB0377 |  | Conserved hypothetical protein (DUF779 domain protein) | 6.0 |
| AB0494 | *ackA1* | Acetate kinase | 31.2 |
| AB0495 | *pta* | Phosphate acetyltransferase | 17.8 |
| AB0501 |  | Sodium:solutesymporter family protein | 4.4 |
| AB0504 |  | Sodium:solutesymporter family protein | 48.8 |
| AB0505 |  | Conserved hypothetical protein (DUF485 domain protein) | 16.4 |
| AB0563 | *soxC* | SulfuroxidationproteinSoxCD, sulfurdehydrogenasesubunit | 12.3 |
| AB0564 | *soxD* | Sulfur oxidation protein SoxCD, diheme cytochrome c subunit | 17.6 |
| AB0565 | *soxX* | Sulfur oxidation protein SoxXA, monoheme cytochrome c subunit | 15.8 |
| AB0566 | *soxY* | Sulfur oxidation protein SoxYZ, sulfur covalently binding protein | 12.1 |
| AB0567 | *soxZ* | Sulfur oxidation protein SoxYZ, sulfur compound chelating protein | 12.7 |
| AB0568 | *soxA* | Sulfur oxidation protein SoxXA, diheme cytochrome c subunit | 18.8 |
| AB0569 |  | Hypothetical protein | 15.3 |
| AB0570 | *soxB* | Sulfur oxidation protein, sulfate thiol esterase | 12.7 |
| AB0572 |  | Conserved hypothetical protein | 4.6 |
| AB0574 |  | Hypothetical protein | 7.2 |
| AB0580 |  | Rhodanese-like protein | 9.0 |
| AB0827 |  | Conserved hypothetical protein | 4.1 |
| AB0828 |  | Outer membrane lipoprotein | 4.3 |
| AB0880 |  | Conserved hypothetical protein | 4.9 |
| AB1064 |  | NADH:flavinoxidoreductase/NADH oxidase | 6.1 |
| AB1066 |  | NAD(P)H-flavinnitroreductase | 5.0 |
| AB1496 |  | Methyl-accepting chemotaxis protein | 4.1 |
| AB1553 | *katG* | Catalase/peroxidase HPI | 9.1 |
| AB1626 |  | Peptidase, M48 family | 4.5 |
| AB2141 |  | DNA-binding ferritin-like protein (Dps/NapA) | 6.2 |
| AB2287 | *sbp* | Sulfate-binding protein precursor | 5.4 |
| **^a^**The functions of the encoded proteins and the AB numbers are indicated according to Miller et al.[15].  **^b^**The fold difference was calculated by comparison of the RNA levels in *A. butzleri* ΔAσ^7^ with those in *A. butzleri* Δσ^7^/Aσ^7^ | | | |
